# Homœopathy, What It Is and What It Is Not

**Published:** 1891-08

**Authors:** 


### BOOK NOTICES.

Homœopathy, What it is and What it is Not. By Thomas Wildes, M. D. Second edition. Jamaica, W. I. Published for the author. Price, 15 cents, for sale at homœopathic pharmacies.

Dr. Wildes, the author of the above work, practiced in New York for a number of years, and then went to the West Indies in a search for health. On arriving in Jamaica he was induced to enter practice there. Opposition on the part of the allopaths induced Dr. Wildes to place before the people of Jamaica the advantages of Homœopathy, and he admirably succeeded, for we are in receipt of cuttings from Jamaica papers in which Homœopathy is defended from the attacks of the allopaths, and in which the establishing of a homœopathic hospital is advocated. Dr. Wildes, in his pamphlet, gives a succinct description of Homœopathy, shows what it is capable of doing, and adds statistics which prove its superiority over the old-school methods.

This pamphlet is calculated to do good missionary work, and we would advise those who are in a community which knows little about Homœopathy to purchase a number for such purpose, and it will also serve even those who are familiar with Homœopathy as a model of how to fight for our principles.

